# Neurodegeneration in acute and chronic central nervous system disorders: Novel ideas and approaches

**DOI:** 10.1002/nep3.69

**Published:** 2024-12-16

**Authors:** Piotr Walczak, Xunming Ji, Shen Li, Johannes Boltze

**Affiliations:** ^1^ Center for Advanced Imaging Research, Department of Diagnostic Radiology and Nuclear Medicine University of Maryland Baltimore Maryland USA; ^2^ Beijing Institute of Brain Disorders Capital Medical University Beijing China; ^3^ School of Life Sciences University of Warwick Coventry UK

Countering neurodegenerative processes remains a major obstacle, although we have witnessed significant recent progress in diagnostic and therapeutic approaches.^1^, ^2^, ^3^ The lack of true tissue regeneration is partially related to the absence of molecular, cellular, and anatomical cues in the adult mammalian central nervous system (CNS) that would allow for recapitulating ontogenesis and thus allow brain tissue regeneration.^4^ This drives the unabated need for better and earlier diagnosis as well as novel cytoprotective approaches in neurodegenerative disorders such as Alzheimer's disease (AD).^5^ Such approaches are covered in the current issue of *Neuroprotection*, which features a mixture of review papers as well as original articles and a clinical trial protocol. The main areas covered are AD and Parkinson's disease (PD), as well as ischemic CNS conditions. This editorial provides a summary of key findings and the most important conclusions reported in these articles.

Blood‐based biomarkers (BBMs) for AD are the primary focus of a review by Liu et al., who highlight the use of BBMs as a screening approach together with cognitive function assessments to select patients for subsequent diagnostic procedures such as lumbar puncture and advanced brain imaging. Although some of these BBMs may target unspecific processes related to AD pathophysiology (e.g., neurofilament light polypeptide to indicate neuronal injury), most of the available BBMs aim to detect pivotal AD pathomechanisms and hallmarks, including the plasma amyloid‐beta (Aβ) 42/40 ratio or phosphorylated tau variants in the blood plasma. Further diagnostic precision can be achieved by considering specific genotypes^6^ such as apolipoprotein E4 expression. Liu et al. also focus on potential biomarkers currently under clinical assessment although those vary considerably regarding diagnostic accuracy. Importantly, the authors also focus on BBM limitations, such as a current lack of standardization regarding sampling, processing, and interpretation, particularly in the context of comorbidities. Multiomics approaches currently under development may provide future diagnostic improvements enabeling more precise and comprehensive AD diagnosis,^7^ ideally also in early or even prodromal stages.

A review paper by Hanumanthappa et al. summarizes recent research in nanoparticles (NPs) as potential drug delivery systems to overcome the blood–brain barrier (BBB). This approach may be advantageous in numerous conditions, including AD and PD. The authors focus on surface‐modified polymer NPs that exhibits better drug absorption and trans‐BBB transport abilities. Next to a focus on NP composition and action, the review provides concise summaries of the BBB anatomy as well as of AD and PD pathomechanisms and established treatment options. This aids less experienced readers. The main part of this comprehensive review contains a detailed description of peptides and proteins used to functionalize NPs for enhanced trans‐BBB transportation in AD or PD, respectively. The review also features a detailed overview of preclinical studies investigating the use of functionalized NPs, including a description of the drug load, route of administration, the disease model, and key experimental findings. The review concludes with a detailed description of three specific NP‐based treatment concepts for AD and PD and a detailed outlook on potential future research directions. Thus, the work by Hanumanthappa et al. underlines the outstanding therapeutic potential of NPs for targeted AD and PD drug therapies.

Khan et al. present a technologically interesting approach aiming at biomarker discovery for PD using mass spectrometry (MS) in a *Drosophila melanogaster* (DM) model system. These insect models only allow the investigation of genetic variants of PD, which are relatively rare in human PD patient populations. However, they allow a very focused and detailed investigation of the consequences of genetic mutations and their impact on dopaminergic neurons. Khan et al. provide a brief overview of PD animal models contrasting toxin‐induced rodent models from genetic DM models. There is also a brief introduction to MS‐based proteomics. The main part of the review focuses on the roles of the most relevant genes in PD as well as key findings obtained in MS‐based studies on these genes using DM models, providing detailed insights into more than 60 preclinical investigations. The review also focuses on potential interactions of PD‐relevant genes, although insights provided by DM‐based models may have limits in that regard.

*Neuroprotection* under ischemic‐hypoxic conditions is the focus of the timely review by Zhao and colleagues. Their main interest is in pre‐ and postconditioning approaches in ischemic stroke based on the concept of hypoxic pockets (HPs). HPs are defined as local volumes of brain tissue that, in contrast to surrounding brain tissue, experience transient hypoxic‐ischemic conditions. Since even brief episodes of hypoxia‐ischemia can have major effects on brain tissue, the local cellular environment, and milieu (e.g., gene expression, metabolic situation, local cytokine, and chemokine factor release) may clearly differ from those in adjacent normoxic areas. After providing a concise introduction to the concept of HPs, Zhao et al. explain the impact of selected interventions and activities, such as isoflurane exposure, exercise, and sensory input, on HPs. The common mechanism between those is that increased local blood flow can reduce size and prevalence of HPs in brain tissue. This may be well in line with neuroprotective effects reported for volatile anesthetics such as isoflurane although detrimental effects can also be observed under specific conditions.^8^ The review then describes how pre‐ and postconditioning can be achieved by isoflurane exposure, exercise but also remote ischemic conditioning in limbs, as well as the therapeutic potential of these experimental interventions in ischemic stroke. The review further provides a detailed outlook on future research directions and makes meaningful comments with respect to potential future clinical implementation of pre‐ and postconditioning approaches.

Thaysen et al. provide a review visiting the old topic of rodent models of ischemic stroke, but with a twist. Given the current need for neuroprotective therapies to work synergistically with recanalization approaches in acute stroke management,^9^ the authors review established rodent stroke models, specifically their advantages and limitations for experimental studies investigating hyperacute acute stroke stages. There is a detailed explanation of the main pathomechanisms in these stages including physiological clot and penumbra formation, excitotoxicity, neuroinflammation, as well as an emerging brain edema against which established rodent stroke models are compared. Thus, the review by Thaysen et al. can serve as a detailed compendium for those wishing to enter this research area. The rich body of literature provided also makes it easy to retrieve additional information, such as methodological details of a respective model.

Chen and coworkers present results from a multivariate Mendelian randomization study investigating a potential list between elevated liver enzymes and various forms of dementia, including AD and vascular dementia (VaD). Genome‐wide association study data of liver enzyme levels and more than 500 single nucleotide polymorphisms (SNPs) were analyzed in a large sample. The liver enzymes investigated comprised alkaline phosphatase (ALP), alanine aminotransferase (ALT), aspartate aminotransferase (AST), and γ‐glutamyl transferase (GGT). SNPs known to relate to hyperlipidemia and alcohol consumption were excluded. There were no associations between ALP, ALT, and AST and any form of dementia, but there was an association between GGT levels and VaD. Although potential causal relations still require more detailed investigation, GGT may serve as both a risk indicator (biomarker) or even a risk factor for VaD, which may inform the development of targeted prevention and treatment approaches.

Moreover, this issue of *Neuroprotection* is reports details of a clinical trial protocol, presented by Yusuying and coworkers. The clinical trial, into which patients are already enrolled, evaluates the safety and preliminary efficacy of a novel device for cerebral venous sinus thrombectomy (CVST) using a prospective, multicenter, and single‐arm objective performance criteria design with a target population size of 146 patients. This is a relevant approach because many thrombectomy devices used for recanalization procedures in large brain‐supplying arteries only have limited applicability in CVST because venous sinuses in the brain exhibit a larger diameter and often a higher clot burden. The protocol paper gives a detailed description of the rationale, the expected patient population, and the clinical approach. Other assets include a very detailed description of the safety and efficacy assessments to be conducted, as well as a detailed sample size calculation.

The current issue of *Neuroprotection* presents highlights from preclinical and clinical research activities tackling neurodegeneration in acute and chronic CNS disorders with a focus on PD, AD, and cerebrovascular conditions. Topics cover the entire research spectrum from biomarker discovery to the evaluation of devices for acute interventions and provide detailed insights into the respective fields. We are happy to present these research highlights that will hopefully serve as comprehensive compendia for those wishing to enter these exciting fields as well as sources of ideas for those who are already established researchers.

## AUTHOR CONTRIBUTIONS

**Piotr Walczak**: Conceptualization; data curation; funding acquisition; project administration; resources; software. **Xunming Ji**: Conceptualization; resources; software; supervision. **Shen Li**: Conceptualization; writing—review and editing. **Johannes Boltze**: Conceptualization; data curation; formal analysis; investigation; writing—original draft; writing—review and editing.

## CONFLICT OF INTEREST STATEMENT

Xunming Ji, Piotr Walczak, and Johannes Boltze are Co‐Editors‐in‐Chief of *Neuroprotection*. They were blinded from reviewing or making decisions on the manuscript. The article was subject to the journal's standard procedures, with peer review handled independently of the Co‐Editors‐in‐Chief and their research groups. Piotr Walczak is a founder and holds equity in IntraArt, LLC and Ti‐com, LLC.
